# Anesthesiological Management of a Sewing Needle Impacted in the Larynx in an Adult: A Case Report

**DOI:** 10.1002/ccr3.72709

**Published:** 2026-05-13

**Authors:** Stefanie R. Senn, Felix C. Jansen, Caveh Madjdpour, Isabel Besozzi, Daniel A. Button

**Affiliations:** ^1^ Department of Anesthesiology Kantonsspital Winterthur Winterthur Switzerland

**Keywords:** aspiration, case report, foreign body, larynx, needle, removal

## Abstract

The anesthesiological management of a sharp object in the upper airway is highly challenging due to the shared high‐risk anatomy. Thorough preparation, including CT scan and fiberoptic assessment, combined with interdisciplinary planning among surgeons, anesthesiologists, and intensive care specialists, enables a structured approach that anticipates complications and ensures safe patient care.

**Abbreviation**
CT scan: computed tomography scan

## 1 Introduction

The ingestion or aspiration of a foreign object is mainly seen in children aged 1–3 years [1]. There is some literature on the ingestion and aspiration of foreign bodies in children and adults [2, 3, 4, 5]. In adults, an intentional or unintentional body impaction occurs, especially in elderly persons or patients with an underlying comorbidity [2]. Esophageal food bolus with or without bone impaction has an estimated annual incidence of 13/100,000 [3]. There are many reports in the literature describing aspiration or swallowing of foreign bodies at the dentist's office, whereby only acute action by the dentist on site is described therapeutically (e.g., Heimlich Maneuver) [4]. Some cases with a primary focus on the circumstances rather than on the exact removal and treatment of the foreign body are described [3, 5]. Impaction of a sharp foreign body in the larynx in adults is a rare clinical entity, with the available literature largely limited to isolated case reports. Comparable cases are present but with completely different procedures after risk stratification [6, 7, 8]. Furthermore, the majority of published cases provide minimal detail regarding anesthetic management, often merely indicating intubation under general anesthesia [7]. However, a sharp needle stuck in the vestibulum of the larynx is a challenging emergency and has not been published like this in the literature before. The most frequently described foreign bodies include herringbone (25%), chickenbone (22.5%) and stapler pins (20%) [5]. Obviously, in such challenging cases, a thorough assessment of the airway before induction of general anesthesia has to be made, and the potential complications due to the removal of the needle (i.e., bleeding, swelling, aspiration of the foreign body into the lower part of the trachea and lungs, infection) have to be anticipated.

## 2 Case History/Examination

A 57‐year‐old, independently living woman with only a history of controlled hypertension accidentally swallowed a 43 mm long and 2 mm thick sewing needle at 2 pm. While sewing at home, the patient had placed the needle between her lips. Initially, she did not even realize the needle impaction and even subsequently had dinner without any complaints. Several hours after the needle ingestion, she developed a sore throat as well as hoarseness and called for an ambulance in the middle of the night. Upon arrival of the ambulance, she was found to be in an agitated state with ataxia and dysarthria. Apart from sinus tachycardia, all vital signs were normal; clinical examination revealed dysarthria but no signs of facial palsy or arm weakness. However, a broad‐based gait was noticed.

## 3 Methods (Differential Diagnosis, Investigations and Treatment)

In line with our stroke protocol, an immediate computed tomography scan (CT scan) with angiography was performed. The CT scan showed a sewing needle stuck in the right vestibulum of the larynx, perforating the ventral part of the larynx in the area of the right glottis with its non‐sharp end remaining in a supraglottic position.

**FIGURE 1:**
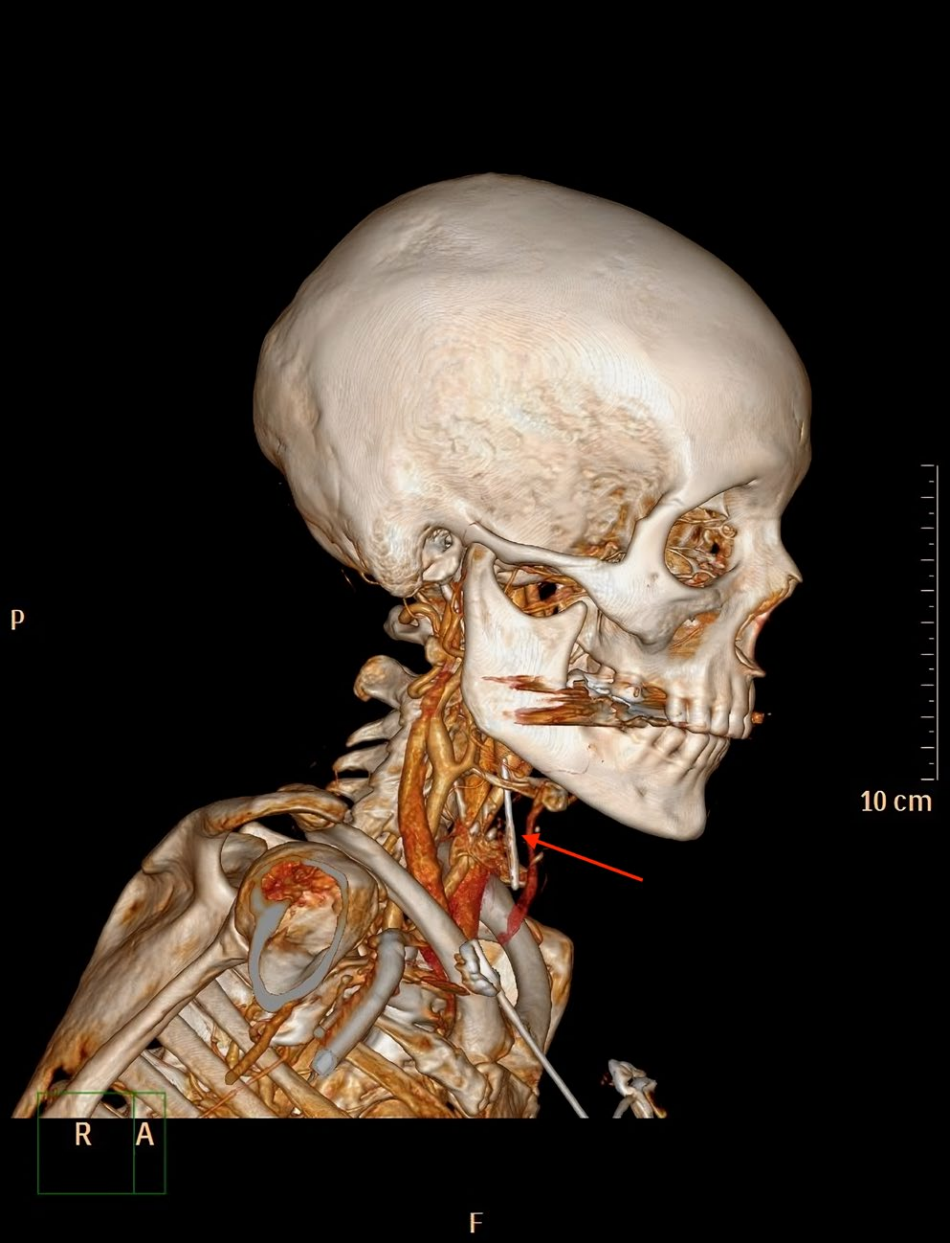
Three‐dimensional reconstruction showing the needle dimension in the upper airway and the proximity to the missed vessels.

**FIGURE 2:**
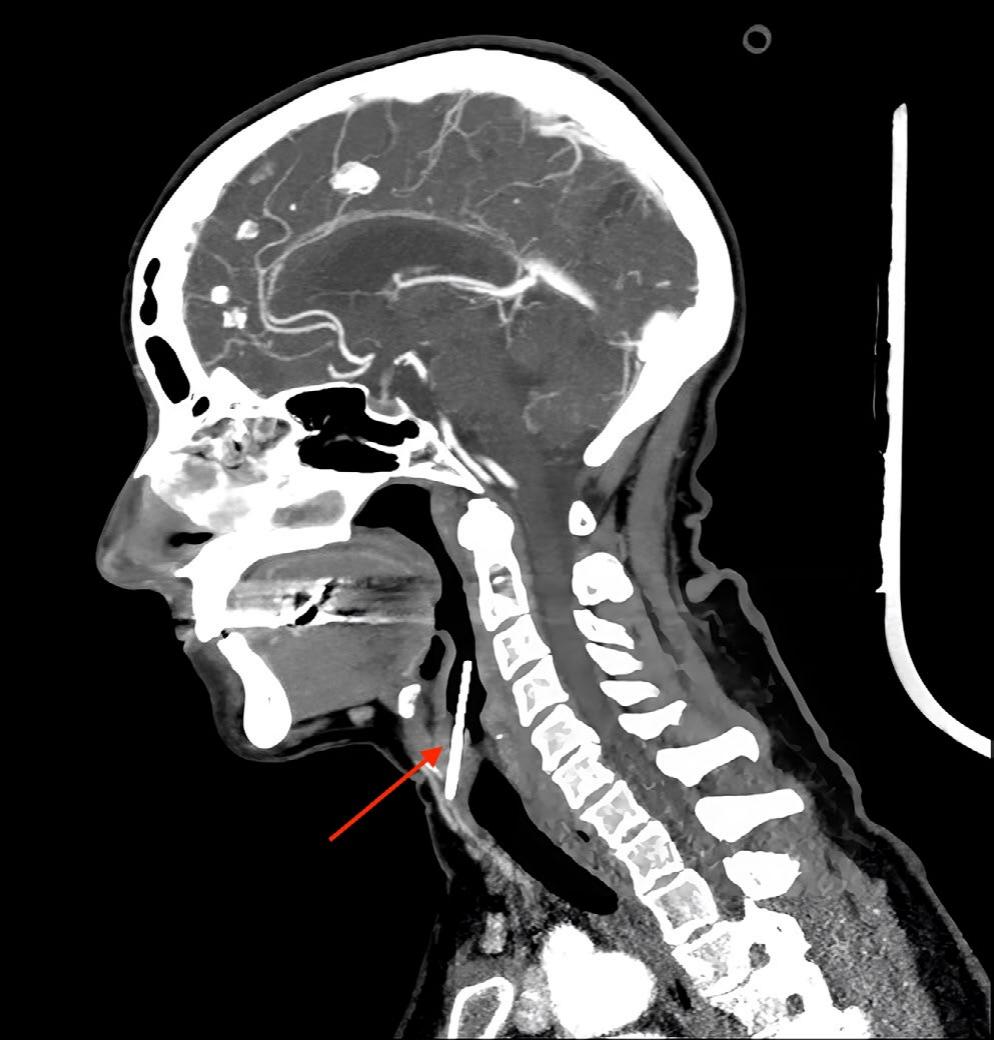
Arterial sagittal computed tomography scan (CT scan) showing the needle penetrating the right vestibular fold, with no evidence of vascular injury.

**FIGURE 3:**
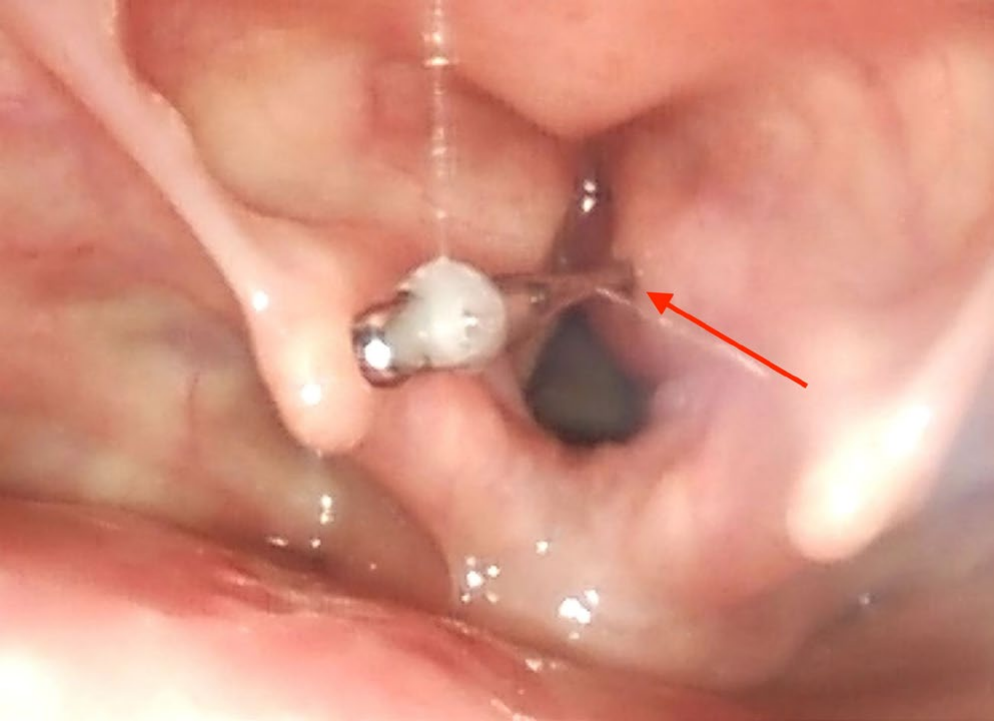
Fiberoptic picture showing the needle stuck in the right vestibular fold of the larynx with its non‐sharp end remaining in a supraglottic position.

The CT scan showed no damage or bleeding due to the needle perforation, although the tip of the needle was in close proximity to vessels of the larynx (Figures 1 and 2). A fiberoptic awake inspection through the nose showed the needle stuck in the right part of the glottis with its blunt end remaining at a supraglottic level. Furthermore, a discrete swelling of the right glottis could be seen while laryngeal functions (i.e., vocal cord function) seemed to be normal (Figure 3).

We decided to remove the needle under general anesthesia with a Magill forceps a C‐MAC videolaryngoscope (C‐MAC Storz) for optimal visualization. Intubation readiness with an endotracheal Magill tube with cuff ID 6 mm with a malleable stylet and the possibility of pharyngeal suctioning using a Yankauer suctioning tip were ensured in case of unexpected hemorrhage, acute swelling, regurgitation, aspiration, or deoxygenation. To avoid losing the needle in the distal airways, we positioned the patient in a supine position.

After informed consent, the patient was sedated with alfentanil 0.5 mg, propofol 100 mg and muscle relaxation with succinylcholine 75 mg. The needle was then removed easily with magill forceps. A repeat fiberoptic inspection of the larynx and the vocal cords showed no bleeding or swelling and the patient emerged several minutes later.

To prevent any swelling, we administered methylprednisolone 2 mg/kg intravenously followed by prednisolone 50 mg per os once a day for 3 days and started an antimicrobial prophylaxis with amoxicillin and clavulanic acid 625 mg per os three times a day for 3 days. Hexetidin 0.1% solution was applied as a topical antiseptic therapy. An intermediate care unit hospitalization for monitoring was initiated overnight.

## 4 Conclusions and Results

Managing the sharp foreign body in the upper airway is highly challenging due to the shared, high‐risk anatomy and potential for catastrophic complications. Careful preparation—including detailed imaging, endoscopic assessment, and interdisciplinary planning—enabled a structured approach and ensured safe management.

Except for the dysarthria, all neurological symptoms disappeared as the patient felt safe and calm. A further detailed patient history showed that dysarthria regularly occurred under stressful conditions and had been known for many years.

In a follow‐up telephone call after discharge, the patient described only an initial hoarseness, which completely resolved within days. The patient subsequently achieved full recovery and the case is considered concluded.

## 5 Discussion

The removal of a sharp object stuck in the delicate region of the larynx is an extremely rare, life‐threatening situation that must be considered and treated individually. In the present case reported, we had to consider the basic complications of penetrating injuries, such as bleeding, swelling or infection, combined with the complications associated with airway management in emergencies (e.g., swelling, aspiration, deoxygenation, secondary damage of mucosa, loss of the airway) and evaluate the best possible approach.

Using various examination techniques (CT scan, fiberoptic pharyngo‐laryngoscopy), we first obtained a precise picture of the problem and anticipated possible complications in order to perform a safe removal of the needle.

Sedation with preserved spontaneous breathing for retrieval of a needle in such a hazardous location represents a significant challenge and requires precise timing and control. Similar approaches have been described, particularly in children but also in adults [1, 6, 7]. Reported indications include a mobile foreign body in the airway, with a risk of dislocation during mask ventilation or intubation, with tracheostomy as the ultimate backup strategy [6, 8]. Other considerations include comorbidities such as obstructive sleep apnea, where maintaining spontaneous breathing with preserved airway tone prevented ventilation problems, or preexisting cardiac or pulmonary conditions, such as pulmonary hypertension, where general anesthesia was preferably avoided [6]. However, it remains a risky maneuver. The patient could have coughed or moved quickly, which would have made the rescue strategy more dangerous. Additionally, the needle was clearly stuck in the mucosa, so we assumed it would not dislocate during mask ventilation. A short anesthesia with relaxation seemed to be the safest method for our patient.

Adequate preoxygenation is essential. In the event of desaturation, intermittent mask ventilation would have been performed for reoxygenation, as the patient had no predictors of difficult mask ventilation or prolonged desaturation. Nasal high flow oxygen could have been an option, particularly as current literature demonstrates favorable results compared with conventional preoxygenation [9, 10]. A further escalation strategy to optimize oxygenation would have been non‐invasive ventilation; however, neither approach was adopted by the team in this case [10]. In a similar future scenario, these strategies would represent reasonable and potentially advantageous options.

Primary intubation—whether conventional, fiberoptic, or awake fiberoptic—was deliberately avoided. The needle was located in a delicate position, and we sought to minimize the risk of distal dislocation caused by manipulation during intubation, as well as the possibility of deeper tissue penetration and additional injury, including tracheal or bronchial wall damage, pneumothorax, or significant bleeding. We also feared damaging the cuff of the endotracheal tube by the needle and the effects of further manipulation of an already irritated mucosa. In the event of an unexpected desaturation not manageable with conventional mask ventilation, we were prepared to proceed with videolaryngoscopy‐guided fiberoptic intubation to ensure optimal visualization, minimize risk, and maximize the likelihood of success [11].

To suppress coughing or patient movements during retrieval, a short‐acting muscle relaxant was administered, allowing removal under controlled conditions. Because the needle had been clearly visualized during fiberoptic examination, we anticipated efficient extraction with Magill forceps during the period of relaxation. Retrospectively, in case of an unexpected event, the use of rocuronium (and the reversal with sugammadex) would have been an even better alternative. The depolarizing agent succinylcholine carries a slightly higher risk of needle dislodgement due to fasciculations and is associated with a broader adverse‐effect profile. Reversal of rocuronium with sugammadex further negates the advantage of the short duration of action offered by succinylcholine.

At this time, the needle had already been in place for 8 h with only minor local effects such as swelling or bleeding. The CT scan also showed no vessels affected by the needle, so we did not expect any major bleeding. We therefore considered the risk of a sudden local problem by removal to be very low compared to the serious risks and side effects of intubation such as dislocation of the needle. The patient was appropriately fasted, with more than 6 h since the last oral intake. In the event of deterioration after removal, emergency intubation equipment was immediately available. In addition, a surgical team was always available in case of unexpected bleeding that could not be controlled by tamponade after intubation or a dislocation of the needle into the deeper airways that could not be evacuated by rigid bronchoscopy [1, 2]. They were also prepared to perform an emergency tracheostomy should intubation prove impossible—for example, due to mucosal swelling or obscured visualization from bleeding.

The procedure was performed with the patient in the supine position to minimize the risk of distal migration of the needle. After removal, immediate fiberoptic assessment of the airway was conducted to detect bleeding, swelling, or any retained foreign objects. We also prophylactically administered methylprednisolone in order to reduce the risk of subsequent edema. Following recovery, the patient was transferred to our intermediate care unit for observation until discharge the next morning.

In summary, a clear and carefully considered strategy formed the foundation for successful management. High‐quality imaging to define the local anatomy—CT scan with angiographic assessment and fiberoptic examination—combined with thorough interdisciplinary discussion, bringing together surgical, anesthetic, and critical care expertise, was essential.

This collaborative approach enabled anticipation of potential complications and ensured safe and effective management of this rare and challenging case.

## Author Contributions

**Stefanie R. Senn:** conceptualization, investigation, writing – original draft, writing – review and editing. **Felix C. Jansen:** conceptualization, investigation, writing – review and editing. **Caveh Madjdpour:** conceptualization, investigation, writing – review and editing. **Isabel Besozzi:** conceptualization, investigation, writing – review and editing. **Daniel A. Button:** conceptualization, investigation, supervision, writing – review and editing.

## Funding

The authors have nothing to report.

## Ethics Statement

The authors have nothing to report.

## Consent

Written informed consent was obtained from the patient for publication of this case report and accompanying images.

## Conflicts of Interest

The authors declare no conflicts of interest.

## Data Availability

The authors have nothing to report.
